# Inherited chromosomally integrated HHV-6A and its potential role in unexplained infertility: the first clinical case from India—a case report

**DOI:** 10.1186/s13256-026-06450-9

**Published:** 2026-08-06

**Authors:** Vani Angra, Arnab Ghosh, Pallavi Sharma, Shalini Gainder, Bharti Joshi, Nalini Gupta, Sunil Sethi, Mini P. Singh, Kapil Goyal

**Affiliations:** 1 Department of Virology, Post Graduate Institute of Medical Education and Research, Chandigarh, 160012 India; 2 Department of Obstetrics & Gynaecology, Post Graduate Institute of Medical Education and Research, Chandigarh, 160012 India; 3 Department of Cytology and Gynaecological Pathology, Post Graduate Institute of Medical Education and Research, Chandigarh, 160012 India; 4 Department of Medical Microbiology, Post Graduate Institute of Medical Education and Research, Chandigarh, 160012 India

**Keywords:** Human herpesvirus 6, Chromosomally integrated HHV-6, Infertility, Reactivation, Case report

## Abstract

**Background:**

Human herpesvirus-6, double stranded DNA virus with 2 variants, has the ability to integrate into the human chromosome, including the germline leading to a condition called as inherited chromosomally integrated HHV-6 (iciHHV-6). This integrated form of HHV-6 (variant A) has been detected in the endometrium and linked to unexplained infertility, but its clinical significance remains unclear. Therefore, it becomes crucial how the virus transitions between the latency and active states in the reproductive tissue. The limited global literature on this topic, highlights the need for further investigation**.**

**Case:**

We describe a case of a 22-year-old nulliparous woman of South Asian ethnicity with a 4-year history of primary unexplained infertility. Routine infertility workup including the hormonal profile, semen analysis, transvaginal ultrasonography and tubal patency assessment were normal. As a part of the research protocol HHV-6 testing was performed, which revealed the presence of HHV-6 DNA in the endometrial biopsy, serum, plasma and PBMCs of the patient. Sequencing results showed the presence of HHV-6A variant, aligning with a variant showing integration at 10q. Droplet digital PCR in the biopsy further confirmed chromosomal integration of HHV-6. Transcriptional analysis detected only latent transcripts with no evidence of active viral replication. Notably, the patient subsequently conceived.

**Conclusions:**

This case suggests a possible association of iciHHV-6-A and infertility; however, a causal relationship cannot be established. Although only latent transcripts were identified at the time of diagnosis, the findings indicate that the presence of iciHHV-6A alone may not be sufficient to impair fertility. Further studies are required to understand the plausible mechanism as to what triggers the reactivation of iciHHV-6 and its further impact on reproductive health.

## Introduction

Human herpesvirus 6 is a double stranded DNA virus belonging to the *betaherpesvirinae* subfamily and genus Roseolavirus. Based on genetic, biological, epidemiological and pathogenic properties, the virus is categorized into two variants, i.e., HHV-6A and HHV-6B [1, 2]. The virus is known to infect various cells and tissues which include liver cells, human fibroblasts, epithelial cells and endothelial cells [3]. A unique attribute of this virus is its ability to integrate both in somatic and germ cells using the telomeric region of the host cell chromosome. In the case of germ line integration, the virus gets integrated into the germ cells of the host. Around 1% of the human population is born with an entire HHV-6 genome inside every cell [4, 5]. This condition is referred to as inherited chromosomally integrated HHV-6A/B (iciHHV-6A/B). Though considered transcriptionally silent, emerging evidence indicates that iciHHV-6 may get reactivated and express viral transcripts and proteins in vivo, potentially influencing host cellular pathways [5].

HHV-6 has been detected in the female reproductive tract including the endometrium and has been linked to unexplained infertility [6]. These observations suggest that HHV-6 may be a potential contributing factor in the pathogenesis of female infertility. Whether HHV-6 remains latent or is actively replicating in the reproductive tissues of women still remains unclear and is crucial for developing treatment strategies for individuals affected by infertility. Herein, we report a case of **inherited chromosomally integrated HHV-6 (iciHHV-6)** in a **nulliparous woman** who later conceived naturally, with detection of only latent transcripts, highlighting the uncertain clinical significance of iciHHV-6 in reproductive health. This case presents a unique opportunity to explore the implications of viral integration in women with unexplained infertility.

## Case presentation

A 22-year-old nulliparous South Asian woman with a 4-year history of primary unexplained infertility presented to the infertility clinic of a tertiary care hospital in North India in November 2023 (Sample ID:SD/99). She was cohabiting with her husband and had no coital difficulties. She had no significant past medical history and surgical history. The patient denied any history of fever or drug allergy.

On general examination, her vital parameters were stable, with a blood pressure of approximately 117/79 mmHg and a body weight of around 72 Kgs. Baseline haematological features revealed mild anaemia (haemoglobin 9.2 g/dL), while total leukocyte count and platelet count were normal. Biochemical parameters including renal and liver function tests as well as serum electrolytes, were within the normal range. Hormonal evaluation showed normal results (TSH, T3, and T4).

**Fig. 1:**
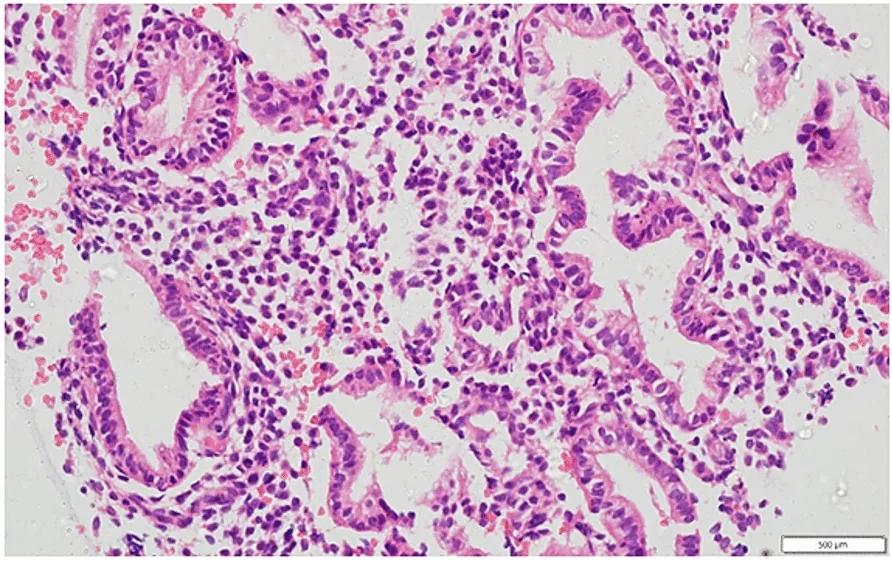
Microphotograph showing endometrium in bleeding late secretory phase with evidence of breakdown (H&E × 20X)

Following baseline evaluation, endometrial biopsy was performed on day 2 of menstruation as part of the institutional sampling protocol for baseline endometrial assessment. The report was histologically normal (Fig. 1). This was followed by a transvaginal ultrasonography and tubal patency assessment which showed no abnormality. The patient was also screened for HIV, hepatitis B (HbsAg) and hepatitis C (HCV), which also came out to be negative. Semen analysis of the partner was within normal limits with adequate sperm count (98 million/ml and motility (approximately 60%).

Based on the above findings the patient was categorized as a case of primary unexplained infertility. The patient was subsequently enrolled for the detection of Human Herpesvirus-6 (HHV-6) as a part of research protocol by taking informed consent. In addition to HHV-6 testing, routine clinical evaluation included screening for other infectious etiologies associated with infertility, such as *Mycobacterium tuberculosis* and *Neisseria gonorrhea* which was negative in this case. On testing the already collected endometrial biopsy for HHV-6, it showed positive results. In addition, the detection of high viral load of HHV-6 in the PBMCs on initial screening raised suspicion for inherited chromosomally integrated HHV-6 (iciHHV-6), which was subsequently confirmed by droplet digital PCR (ddPCR).

## Diagnostic findings

### Investigations

A part of the endometrial biopsy was transported to the Department of Virology for screening for Human Herpesvirus-6. DNA extraction was done using the Qiagen DNA Blood and Tissue Extraction Kit (Qiagen, Germany) and was subjected to conventional nested PCR, targeting two specific genes: the U67 gene, which codes for the major capsid protein, and the U94 gene, a marker of viral latency. In addition, blood samples (plasma, serum, peripheral blood mononuclear cells), were collected for HHV-6 quantification through real-time PCR. Positive blood and biopsy samples were further analyzed by Sanger sequencing, to determine the HHV-6 variant. Chromosomal integration was confirmed by droplet digital PCR (Bio-Rad). mRNA studies were conducted to examine the transcriptional activity of the virus, targeting the active U67 gene (indicating active/lytic infection) and the latent U94 gene (indicating viral latency).

## Results

**Fig. 2:**
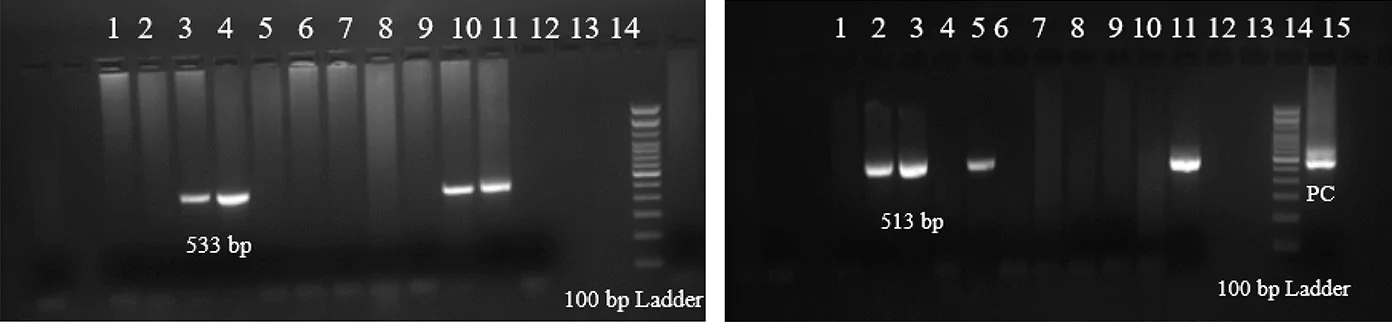
**a** HHV-6 conventional Nested PCR for HHV-6 DNA targeting ORF-U94. **b** HHV-6 conventional Nested PCR for HHV-6 DNA targeting ORF-U67

All the samples including the endometrial biopsy, serum, plasma and PBMCs of this patient tested positive for HHV-6 (Fig. 2a, b). The PBMC’s viral load (5.5 log_10_ copies/ml) was consistent with inherited chromosomally integrated HHV-6 (iciHHV-6), reinforcing the diagnosis of viral integration in the patient. Droplet digital PCR analysis of the endometrial biopsy revealed a viral load of 958 copies/µl, with HHV-6 copies in the biopsy to be 0.96 copies/cell, confirming inherited chromosomal integration [5]. Sanger sequencing identified HHV-6A as the major variant both in the endometrial biopsy and PBMCs **(Accession No. PV641051), showing a 100% nucleotide sequence identity to a reference sequence at GenBank corresponding to a chromosomal 10q integration**. The transcriptional analysis detected only latent transcripts with no active transcripts in the endometrial tissues sample.

## Discussion

The case we present here adds to the growing body of evidence indicating that HHV-6 can be present in the endometrial tissue and other reproductive tissues [7]. The findings in this case raise several important questions regarding the role of HHV-6 in infertility, its mechanism of persistence, and whether it actively or passively influences reproductive outcomes. According to the already cited literature it is proposed that the viral infection of the endometrial natural killer cells, particularly the uterine natural killer cells is one of the contributing factors to the pathogenesis of primary infertility. However, the current study does not provide direct evidence of active viral involvement in infertility.

Detection of HHV-6A well aligns with study by Marci et al., 2016, where only HHV-6A was found in the endometrium epithelial cells of women with unexplained primary infertility, which further supports the association of this variant with reproductive dysfunction [8]. However, it is important to note that these observations remain correlative and do not establish causality.

Though in this case the patient diagnosed with iciHHV-6, eventually conceived naturally, suggesting that the virus reactivated earlier potentially causing local inflammation and impairing infertility and then returning to latency at the time of investigation, such a mechanism cannot be confirmed by the study. The absence of active transcripts (U67), with detection limited to U94, raises important questions regarding viral activity; however, this observation should be interpreted with caution due to technical limitations of mRNA analysis, including RNA instability, low copy number of HHV-6 transcripts, and their transient expression. At present the precise triggers for in vivo reactivation of iciHHV-6 is still unknown, but experimental evidence suggests that it can be induced to a state of lytic replication. However, recent studies have started to report human genomes carrying only one portion of the HHV-6B genome, supporting the idea of in vivo excision and possible viral reactivation [9]. However, these observations underscore the need for caution before attributing a direct pathogenic role to iciHHV-6.

The viral load detected in the endometrial biopsy and the presence of approximately 1 copy per further suggests inherited chromosomal integration, and raises questions about the complexities of understanding the mechanism of action of iciHHV-6. Though latent integration may appear clinically non-significant; however, reactivation or even transient nature of the virus may affect reproductive outcomes.

This case emphasizes the need for further research to better understand the clinical implications of inherited chromosomally integrated HHV-6, in women with unexplained infertility. The study emphasizes the need for more research on the role of iciHHV-6 in the endometrium, particularly regarding reactivation dynamics and its potential contribution to infertility. At present, a routine screening for HHV-6 cannot be recommended based on a single case. However, evaluation can be considered in cases of infertility, where the conventional etiologies are excluded.

## Conclusion

This case highlights the detection of iciHHV-6A in the endometrial epithelial of a woman with unexplained infertility. However, given that the virus was identified in a latent, integrated form and the patient subsequently conceived, the findings suggest an association rather than a causal relationship.

Further large-scale longitudinal studies are required to determine the prevalence, reactivation potential, and clinical significance of iciHHV-6 in infertility. In addition, distinguishing the roles of HHV-6A and HHV-6B in reproductive health remains an important area for future investigation. Though a single case, cannot establish the clinical significance of HHV-6. However, this report highlights the importance of considering HHV-6 as a potential factor in selected cases of unexplained infertility, where all other causes have been excluded. The report also highlights the utility of molecular techniques such as PCR and ddPCR in identifying iciHHV-6. However, given the limited evidence and high cost and limited availability restrict their routine use in standard clinical settings.

## Data Availability

All data generated or analysed during this study are included in this published article.

## Abbreviations

HHV-6: Human herpesvirus-6
iciHHV-6: Inherited chromosomally integrated HHV-6
